# Dynamic Tracking of Biological Processes Using Near-Infrared Fluorescent Single-Walled Carbon Nanotubes

**DOI:** 10.1021/acsami.4c10955

**Published:** 2024-10-08

**Authors:** Srestha Basu, Adi Hendler-Neumark, Gili Bisker

**Affiliations:** † Department of Biomedical Engineering, Faculty of Engineering, Tel Aviv University, Tel Aviv 6997801, Israel; ‡ Center for Physics and Chemistry of Living Systems, Tel Aviv University, Tel Aviv 6997801, Israel; § Center for Nanoscience and Nanotechnology, Tel Aviv University, Tel Aviv 6997801, Israel; ∥ Center for Light-Matter Interaction, Tel Aviv University, Tel Aviv 6997801, Israel

**Keywords:** single-walled carbon nanotubes, near-infrared fluorescence, dynamic tracking, cells, tissues, organisms

## Abstract

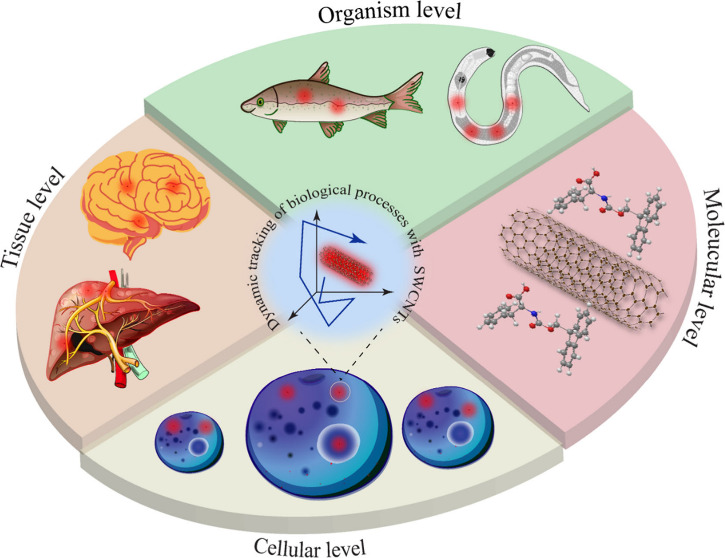

Biological processes are characterized by dynamic and elaborate temporal patterns driven by the interplay of genes, proteins, and cellular components that are crucial for adaptation to changing environments. This complexity spans from molecular to organismal scales, necessitating their real-time monitoring and tracking to unravel the active processes that fuel living systems and enable early disease detection, personalized medicine, and drug development. Single-walled carbon nanotubes (SWCNTs), with their unique physicochemical and optical properties, have emerged as promising tools for real-time tracking of such processes. This perspective highlights the key properties of SWCNTs that make them ideal for such monitoring. Subsequently, it surveys studies utilizing SWCNTs to track dynamic biological phenomena across hierarchical levels—from molecules to cells, tissues, organs, and whole organisms—acknowledging their pivotal role in advancing this field. Finally, the review outlines challenges and future directions, aiming to expand the frontier of real-time biological monitoring using SWCNTs, contributing to deeper insights and novel applications in biomedicine.

## 1 Introduction

Biological processes exhibit intricate and dynamic temporal patterns due to the complex interplay of genes, proteins, and cellular components, which respond to various internal and external stimuli.^1,2^ This complexity originates at the molecular level, involving DNA, RNA, proteins, and enzymes, and extends through cellular, tissue, and organ systems, ultimately involving the entire organism. Key biological processes such as metabolism, cell division and growth, gene expression and regulation, homeostasis, and immune response are interconnected and often influence each other.^3^ This interconnectivity creates a dynamic and responsive system that is essential for the survival and adaptation of living organisms. Therefore, real-time dynamic tracking of these biological processes is crucial for a comprehensive understanding of the intricacies inherent in living systems. It enables the observation of how these processes evolve and respond to various stimuli over time, providing insights into the fundamental mechanisms of life.^4^ Moreover, real-time tracking is essential for early diagnosis of disease progression, paving the way for targeted therapeutics, drug development, and personalized medicine. By monitoring these processes in real-time, researchers and clinicians can detect abnormalities early, tailor treatments to individual patients, and develop more effective therapeutic strategies, ultimately improving patient outcomes and advancing healthcare.^5,6^

A promising class of optical probes for the live tracking of biological processes includes fluorophores such as organic dyes, quantum dots, carbon dots, and inorganic complexes. However, monitoring biological processes using such conventional spectroscopic probes is challenging due to the dominant presence of proteins, nucleic acids, and other biologically active molecules within living organisms that often absorb, scatter, or autofluoresce in the visible spectral region, causing significant interference.^7,8^ Real-time monitoring of biological processes in the near-infrared (NIR) region is highly beneficial.^9−11^ NIR optical probes provide real-time, spatiotemporal information as biological samples are mostly transparent in this region, where absorption, scattering, and autofluorescence are minimal compared to the visible range.^12−17^ This allows for deeper tissue penetration and reduced background interference, enabling clearer and more precise tracking of biological processes.^18^ Consequently, dynamic processes at the molecular, cellular, and systemic levels can be observed, enhancing our understanding of complex biological systems and improving disease diagnosis and treatment. To this end, NIR fluorescent single-walled carbon nanotubes (SWCNTs) are particularly advantageous due to their intrinsic physicochemical and optical properties.^19,20^ SWCNTs can be considered as rolled-up graphene and are characterized by their chiral index, denoted by (*n,m*), where *n* and *m* are integers that define the structure of the carbon lattice. This chiral index essentially describes how the nanotube is “rolled up” along vector **c**, which is defined as **c** = *n***a**_**1**_ + *m***a**_**2**_, with **a**_**1**_ and **a**_**2**_ being the lattice vectors of the graphene. The chiral vector not only dictates the diameter of the resulting SWCNT, which can vary from approximately 0.4 nm to ∼2 nm, but also significantly influences its electronic properties. When *n* - *m* = 0 (armchair configuration), the nanotube exhibits metallic behavior; when *n* – *m* = 3*j* (where *j* is an integer), it is semimetallic; and for all other (*n,m*) values, it is semiconducting. In semiconducting SWCNTs, light absorption generates excitons, which can radiatively decay in the form of fluorescence emission in the NIR range, specifically at wavelengths greater than 870 nm.^10^ This NIR fluorescence is of considerable interest for applications in biological imaging due to its deeper tissue penetration and lower background interference^10,19,21^ making them ideal sensors for monitoring essential biomarkers such as proteins,^22−30^ pathogens,^31,32^ small molecules,^33−37^ oncometabolites,^38^ hormones,^39−41^ volatiles,^42,43^ lipids,^44−46^ sugars,^47−51^ neurotransmitters,^10,14,52−56^ microRNA,^57,58^ lysosomal pH,^59^ and enzymatic activity and inhibition.^60−68^ Their lack of photobleaching or blinking is an additional advantage over common fluorophores, and fluorescent proteins or dyes.^69^ Moreover, SWCNTs, suspended with appropriate corona phases, are known for their biocompatibility and long-term stability in biological environments.^70−73^ Surface functionalization of SWCNTs with hydrophilic groups, such as hydroxyl, carboxyl, or amine, significantly improves their dispersibility in aqueous solutions, as well as their stability and biocompatibility.^70^ By grafting biocompatible polymers like poly(ethylene glycol) (PEG) onto these functionalized SWCNTs, oxidative stress on cells is reduced and cytotoxicity is mitigated. Noncovalent coatings with biocompatible materials offer additional protection, preventing SWCNT aggregation and further decreasing cytotoxicity. These enhancements allow CNTs to effectively carry drugs, targeting ligands, and genes, thus expanding their potential in biomedical applications.^70^

For instance, PEG-coated SWCNTs have shown extended circulation times in the bloodstream, making them highly effective in bioimaging applications.^71^ DNA-wrapped SWCNTs have also demonstrated excellent stability and biocompatibility with no adverse cellular responses. Studies have shown that the pharmacokinetic profile and biodistribution of SWCNTs can be favorably modulated by appropriate surface coatings, with findings indicating liver specificity and significant signal attenuation within 14 days in mice. Despite their persistence at low levels in various organs, comprehensive assessments of organ function and overall health suggest that these SWCNTs are both short- and long-term biocompatible, making them promising tools for preclinical research.^71^

On an industrial scale, SWCNTs can be efficiently produced through chemical vapor deposition of methane,^74^ and large-scale separation of single-chirality SWCNTs can be achieved using simple methods like gel chromatography.^75^ Additionally, large-scale functionalization of SWCNTs is possible using reactions such as the Billups-Birch reduction,^76^ or shear force mixing.^77^ Furthermore, the typically low quantum yield of SWCNTs can be significantly enhanced to as high as 62% by coupling them with plasmonic nanocavity arrays, which greatly improves their light emission properties and opens up new possibilities for photonic applications.^78^

Due to these attributes, SWCNTs have been used to track various biological processes from the molecular level to the whole organism.^79−89^ For example, SWCNTs have enabled real-time tracking of fibrin-clot formation, providing insights into the coagulation cascade at the molecular level.^79^ At the cellular level, SWCNTs have been used to monitor cytoskeletal dynamics,^83^ while at the organ level, they have been used to probe liver fibrosis prognosis by tracking changes in the interstitial space diffusive microenvironment.^85^ At the organism level, SWCNTs have been utilized to track gastrointestinal contraction and feeding mechanisms in *Caenorhabditis elegans* (*C. elegans*) nematodes.^86,87^ Importantly, in many of these (and other) examples, tracking biological events with SWCNTs has been complemented by estimating biologically relevant parameters, both qualitatively and quantitatively.^83^ Thus, in identifying the critical advancements in tracking biologically relevant processes achieved using SWCNTs, it is important to take a deeper view into the relevant techniques employed to achieve this and understand the essential features of these studies.

**Scheme 1:**
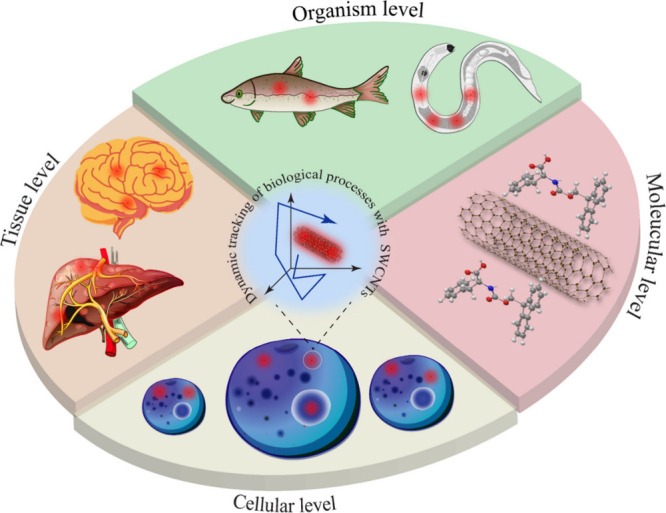
Real-Time Tracking of Biological Processes at the Molecular, Cellular, Tissue, and Organism Levels Using NIR Fluorescent SWCNTs

In this review, our aim is to highlight the physicochemical attributes of SWCNTs that render them preferable for real-time monitoring of biological processes compared to conventional spectroscopic probes operating in the visible spectral region. Next, we will delve into the various strategies employed for customizing the functionalization of SWCNTs to enhance their suitability for this purpose. Emphasizing the uniqueness of each study, we will further highlight the significant research endeavors in this field that have propelled it forward (Scheme 1). Finally, we will identify some of the remaining challenges in this domain and propose potential solutions to further advance the field. We intend to emphasize here that this review focuses specifically on studies highlighting the dynamic tracking of SWCNTs to extract real-time information about biological processes rather than imaging biological samples. This distinction highlights the unique capability of SWCNTs to provide dynamic insights into physiological changes and disease progression within complex biological environments.

## 2 Suitability of SWCNTs as Probes for Tracking Biological Processes

Comprehending the core characteristics of SWCNTs that make them suitable for tracking biological events is of paramount importance. Not only does it allow for further harnessing their application potential but also for better contextualization of their appropriateness in the studies conducted thus far.

The biological compatibility of a probe is a crucial prerequisite for its effectiveness in tracking biological events. Given that SWCNTs are extended π-systems, they are inherently hydrophobic and tend to aggregate in aqueous environments. Therefore, a critical step in utilizing SWCNTs as probes for biological tracking is achieving their stable dispersion in water.^10^ In this context, noncovalent surface functionalization has proven effective in dispersing SWCNTs in aqueous media. Specifically, biopolymers like DNA and RNA can form π–π interactions with the SWCNTs via their nucleobases, thus exposing their negatively charged phosphate backbones, which solvate the SWCNT–nucleic acid complex.^90,91^ Additionally, amphiphilic biopolymers such as proteins,^50^ lipid-polymers,^12^ and peptides^92^ have been successfully employed to suspend SWCNTs in water, further enhancing their biological compatibility. This, in turn, ensures their suitability for real-time tracking of biological processes.

Optical probes used for probing living systems at the microscale should have appropriate dimensions that match the size of biological molecules and cellular components.^93^ In this regard, SWCNTs are particularly well-suited as optical probes. SWCNTs typically have lengths ranging from a few hundred nanometers to several tens of micrometers and diameters on the nanometer scale. These dimensions enable SWCNTs to be readily observed and tracked using optical microscopy techniques.^93,94^

Next, in the context of tracking live biological events, it is highly desirable that the probes operate in a region where the biological species themselves are transparent and background-free—meaning that scattering, absorption, and autofluorescence are reduced in that particular region.^95^ In such cases, the information obtained using such probes is devoid of background interference, leading to clearer and more accurate observations of biological processes. Notably, SWCNTs are well-suited for this purpose as they fluoresce in the NIR region, which coincides with the biological transparency window.^95,96^ In this spectral range, biological tissues exhibit minimal autofluorescence and absorption, allowing for deeper tissue penetration and reduced background interference during the dynamic tracking of biological events. As a result, SWCNT-based optical probes offer excellent signal-to-noise ratios and enable the high-resolution tracking of biological events in real-time.

In the next level, fluorescence-based probes for tracking “life in motion” are desired to maintain stable photophysical properties for the duration of the biological event studied. This stability ensures reliable data collection over extended periods, facilitating the accurate monitoring of dynamic processes without signal degradation. Probes with high photostability are thus crucial for the effective real-time tracking of biological events. SWCNTs meet this requirement as well, with their minimal photobleaching and blinking for prolonged visualization in complex biological settings.^72,97^

In addition to their optical properties, it is crucial that the probes also maintain their physicochemical stability, even under extreme biological conditions. This stability ensures that the probes remain functional and effective in various physiological environments, including those with fluctuations in the pH, temperature, and ionic strength. Moreover, it is desirable for optical probes to be biocompatible. This means they should be noncytotoxic and should not induce adverse effects on the biological species of which they are used to study. In that regard, the well-defined surface of SWCNTs, which allows for their suspension with a variety of corona phases, addresses both the aforementioned issues and helps them remain both biocompatible^70,71^ and physicochemically stable under biological conditions.^92,98^ The versatility in functionalizing the surface of SWCNTs enables the attachment of different molecules, such as biopolymers, lipids, or peptides, forming a corona that enhances their stability and biocompatibility.^99^ These corona phases can be tailored to protect the SWCNTs from degradation or aggregation, ensuring that their optical properties and structural integrity are maintained, even under extreme biological conditions. This dual functionality—achieving physicochemical stability and biocompatibility—makes SWCNTs highly suitable for real-time tracking of biological events, providing accurate and reliable data without compromising the health of the biological species being studied.

Due to the aforementioned virtues, SWCNTs have emerged as a popular choice for real-time tracking of a plethora of biologically relevant processes, which forms the core of our subsequent discussion.

## 3 SWCNTs for Dynamic Tracking of Biological Processes

At this juncture, it becomes evident that SWCNTs emerge as promising candidates for dynamically tracking intricate biological processes. The subsequent objective involves exploring diverse instances documented in the literature where SWCNTs have been employed to investigate biologically significant processes spanning from the molecular scale to that of an entire organism.

### 3.1 At the Molecular Level

The complexity and continuous evolution of biological processes stem from their molecular origin, which involves essential biomolecules such as amino acids, peptides, proteins, DNA, and RNA, among others.^100^ These molecules serve pivotal roles in various cellular functions, encompassing cell signaling, metabolism, gene expression, and the storage and transmission of information.^101^ The dynamic interplay among these biomolecules, both intra- and intermolecular, lies at the heart of living organisms’ functionality. For example, proteins interact with other proteins, nucleic acids, and small molecules to form involved networks that govern cellular activities and respond to environmental cues.^102^ Meanwhile, DNA and RNA molecules encode genetic instructions and partake in processes like transcription, translation, and gene regulation, ultimately dictating an organism’s phenotype and behavior.^103^ Furthermore, these biomolecules frequently undergo post-translational modifications, alternative splicing, and other regulatory mechanisms, which further enhance the complexity and diversity of biological processes. These molecular interactions and modifications shape the convolutions of biological systems, enabling organisms to adapt to changing environments and undergo evolutionary changes over time. Therefore, real-time tracking of these molecular interactions, which define the core of crucial biological processes, is of paramount importance. NIR fluorescent SWCNTs have been utilized for this purpose, allowing researchers to monitor these interactions in real-time.

The fundamental approach in this endeavor involves suspending SWCNTs with biologically relevant biomolecules and monitoring their transformation into a product as part of the biological process. The feasibility of this strategy stems from the well-defined and interactive surface of SWCNTs, which can be rationally functionalized with a wide array of biomolecules. These biomolecules may form either the primary or the secondary corona phases around the SWCNTs. The appropriately corona-encased SWCNTs are then typically exposed to a third (bio)chemical agent, which orchestrates the transformation of the corona into the product. Consequently, the key characteristics of the SWCNTs, such as diffusion kinetics and optical properties, undergo changes, facilitating the tracking of that particular process. A critical requirement of this approach for “live tracking” of the transformation process is ensuring that the SWCNTs remain attached to the reactive units throughout the reaction. Additionally, the subsequent transformation of these units into the product must be directly transduced into optical signals by the SWCNTs.

**Figure 1:**
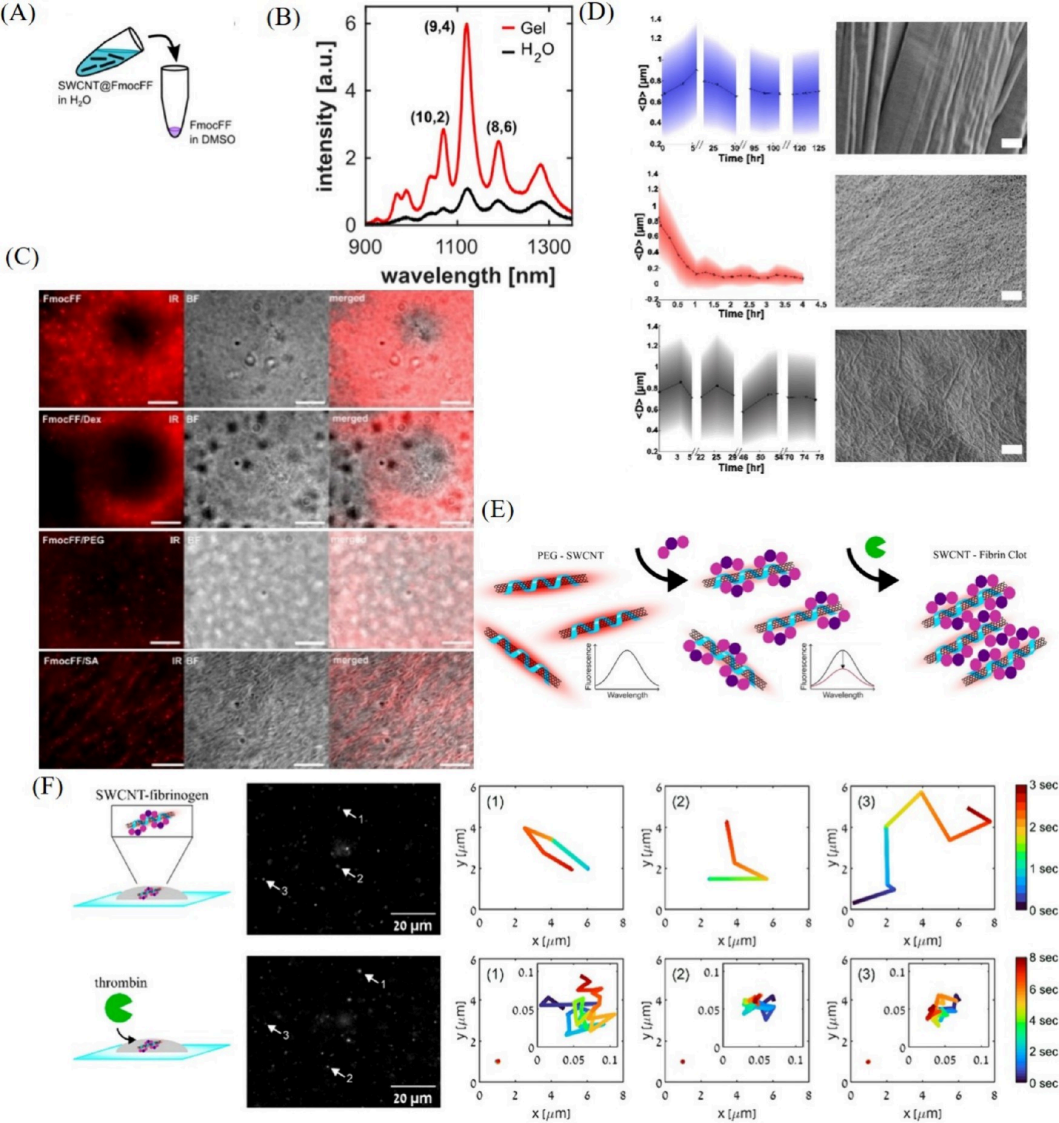
(A) Schematic depicting the solvent-switch concept for SWCNTs@Fmoc-FF in order to initiate gelation. (B) Fluorescence emission spectra of SWCNTs@Fmoc-FF in water following the solvent switch. (C) NIR fluorescence imaging of SWCNTs within Fmoc-FF and Fmoc-FF/polymer hydrogels acquired 1 h postgelation, illustrating the morphology of the hydrogels. The left column displays NIR channel images, the column panel presents bright field images, and the right column shows the merged images. Scale bar is 20 μM. Reproduced with permission from ref (80). Copyright 2022 American Chemical Society. (D) The left column shows the time-dependent mean displacement of SWCNTs within Fmoc-phenylalanine (blue), Fmoc-tryptophan (red), and Fmoc-tyrosine (gray) hydrogels, and the right column shows their corresponding morphology as observed under SEM. Reproduced with permission from ref (104). Copyright 2024 Elsevier. (E) Schematic illustrating the concept of real-time monitoring of fibrin clot formation from fibrinogen appended to PEG-SWCNTs, following the addition of thrombin. (F) Time-resolved NIR single-particle tracking of DPPE-PEG-SWCNTs (indicated by white arrows) cast on a microscope slide, illustrating their trajectories before and after thrombin addition. Thrombin, when added to fibrinogen-appended DPPE-PEG SWCNTs, induces the formation of fibrin clots, which subsequently slow their diffusion. Reproduced with permission from ref (79). Copyright 2023 American Chemical Society.

In this context, the Bisker research group has successfully demonstrated the use of SWCNTs as probes to track the dynamic self-assembly of peptides into hydrogels, which closely resemble the morphology of fibrous proteins in the extracellular matrix.^80^ SWCNTs are ideal for examining the real-time gelation of peptides into hydrogels due to two main factors: (1) their long, fibrillary, and flexible structure closely mimics the fibrous peptide self-assemblies that form hydrogels and (2) changes in the local environment of SWCNTs directly influence their optical properties. To achieve this, SWCNTs were suspended with Fmoc-diphenylalanine (FmocFF), a compound well-known for forming self-supporting hydrogels. A “solvent-switch” was then performed to initiate the assembly process (Figure 1A). This process was directly observed spectroscopically, showing an increase in the fluorescence of FmocFF-SWCNTs (Figure 1B). Additionally, microscopic techniques were used to study the morphological evolution of the gelation process (Figure 1C). (Figure 1As shown in Figure 1C, SWCNTs embedded in FmocFF and FmocFF-polymer hybrids were effectively used to stain the corresponding hydrogels, revealing their morphological features, such as nucleation sites in the FmocFF and FmocFF/Dex gels, and a fibrillary structure in FmocFF/SA. Single particle tracking of the SWCNTs in short videos taken at different times following the gelation could also be used to probe the evolution and the modulations of the gels’ microenvironment over time.^80^ Even after 5 h of gelation, SWCNTs continued to diffuse within the gels, despite rheological measurements indicating that the mechanical properties of the gels had stabilized by this point. However, after 24 h of gelation, the SWCNTs exhibited no further diffusion, appearing fully immobilized within the gel matrix. A recent study also demonstrated real-time monitoring of hydrogel formation using SWCNTs based on various Fmoc aromatic amino acids such as tyrosine, tryptophan, and phenylalanine.^104^ Single-particle tracking of SWCNTs within these hydrogels was employed to monitor the gelation process in real-time. The study revealed that in Fmoc-tyrosine and Fmoc-phenylalanine hydrogels, the mean displacement of SWCNTs remained stable over time. In contrast, in Fmoc-tryptophan hydrogels, the mean displacement of SWCNTs notably decreased within the first hour of gelation before stabilizing. Interestingly, the consistent displacement of SWCNTs in Fmoc-tyrosine and Fmoc-phenylalanine hydrogels correlated with their observed morphological structures under scanning electron microscopy (SEM), showing fibrillary assemblies even before gelation began (Figure 1D). This observation suggests that these structures did not impose additional constraints on the SWCNT displacement upon gelation. Conversely, the gradual reduction in the displacement of Fmoc-tryptophan SWCNTs upon gelation indicated a more restricted microenvironment, possibly due to smaller pore sizes within the gels, a finding further supported by SEM analysis. This method of probing the real-time morphological evolution of peptide hydrogels through dynamic self-assembly using noninvasive optical probes has paved the way for real-time monitoring of diseases characterized by the assembly of peptides and proteins, such as Alzheimer’s disease.

In another study, it was demonstrated that SWCNTs functionalized by polyethylene glycol (PEG), to which fibrinogen was adsorbed, could be used as probes for real-time tracking of fibrin clot formation.^79^ This was achieved by observing the variation in the diffusion rates of SWCNTs before and after the addition of thrombin, which converts fibrinogen to insoluble fibrin, thereby initiating the clotting process. This study is significant, as the conversion of fibrinogen to fibrin is one of the final steps in the blood coagulation cascade. Specifically, SWCNTs were suspended with dipalmitoylphosphatidylethanolamine-polyethylene glycol (DPPE-PEG). In the next step, fibrinogen was attached to the DPPE-PEG-suspended SWCNTs. Thereafter, thrombin was added to initiate the transformation of fibrinogen (attached to the SWCNTs) into insoluble fibrin and the polymerization of monomeric fibrin into a fibrin clot (Figure 1E). A set of experiments was conducted within a droplet cast on top of a microscope slide (Figure 1F, left panel), where the trajectories of three independent SWCNTs (Figure 1F, middle panel) were followed before and after the addition of thrombin (Figure 1F, right panel). Interestingly, the addition of thrombin led to a significant reduction in the diffusion rate of these particles from 8.9 ± 7.8 μm^2^ s^–1^ to 2.4 × 10^–4^ ± 8.1 × 10^–5^ μm^2^ s^–1^. Furthermore, the diffusion of SWCNTs within the fibrin clots was found to slow as the clot formation progressed, providing a direct and quantitative measure of the rate of clot formation. This slow-down of the diffusion was shown to depend on both the fibrinogen and thrombin concentrations, providing quantitative information on both these coagulation factors.^79^ Importantly, this approach not only offers insights into the dynamics of blood coagulation but also holds promise for the early diagnosis of diseases associated with coagulation abnormalities. By leveraging SWCNTs as probes for real-time monitoring of clot formation, clinicians may be able to detect clotting disorders and thrombotic events at an early stage, enabling timely intervention and improved patient outcomes.

### 3.2 At the Cellular Level

The intricacy of biological systems, originating at the molecular level, as discussed in the previous section, expands to the subcellular and cellular levels through the organization of molecular components into specialized structures. These structures include organelles such as the nucleus, mitochondria, endoplasmic reticulum, and Golgi apparatus as well as macromolecular complexes like ribosomes and spliceosomes. Therefore, the real-time tracking of biological events at the cellular level holds significant importance in unraveling dynamic behaviors and regulatory mechanisms within living cells. This endeavor fosters an enhanced comprehension of fundamental biological processes and their implications for both health and disease.

In this context, SWCNTs have proven to be valuable tools for investigating numerous important cellular phenomena. However, it is imperative to exercise caution when using SWCNTs for such purposes. Ensuring their efficacy involves functionalizing them with suitable corona phases, a crucial step in preserving their optical properties over extended periods. Additionally, this functionalization process must render SWCNTs biocompatible to prevent any adverse effects on the cellular health.

**Figure 2:**
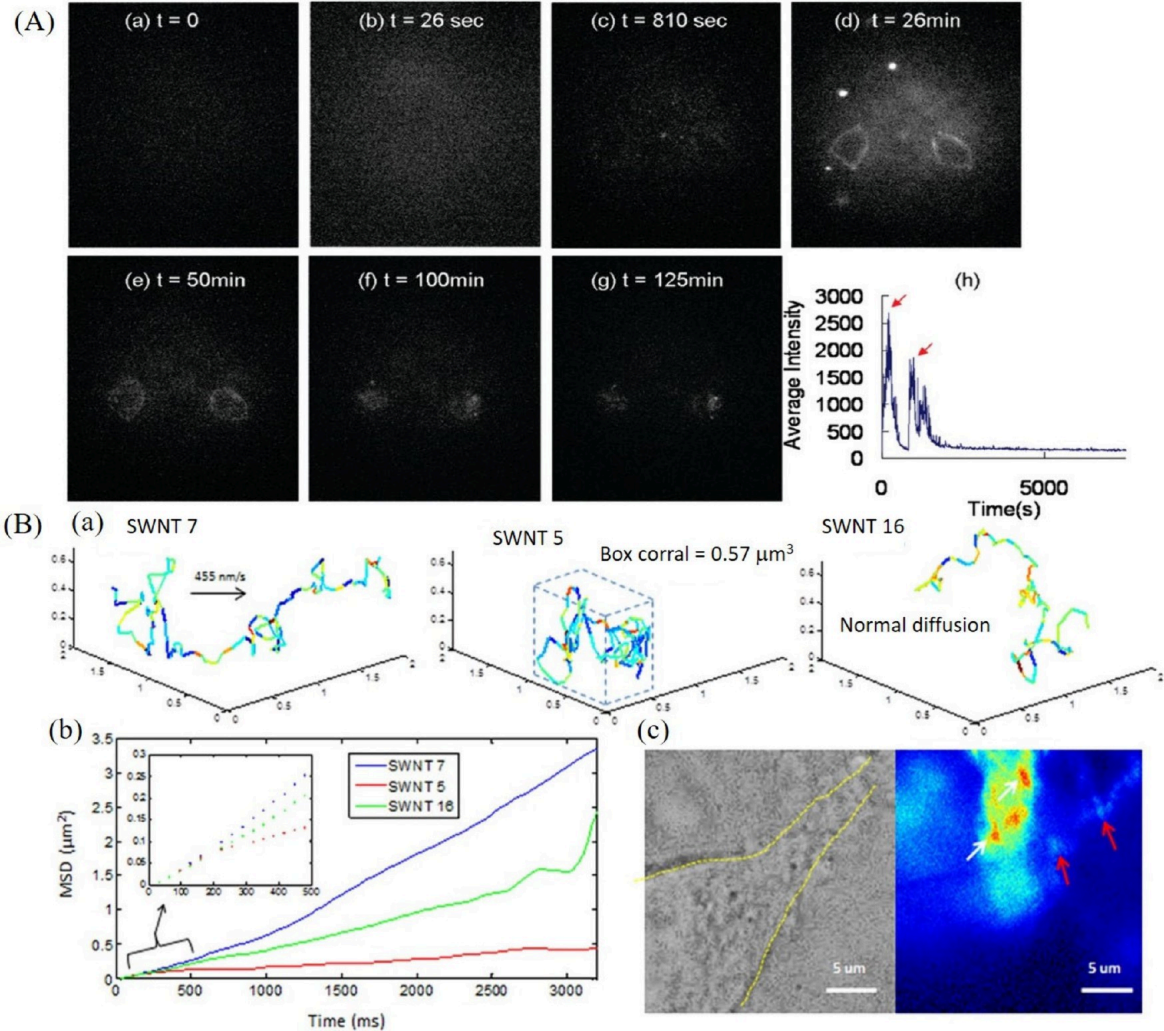
(A) Time-dependent perfusion of DNA-SWCNTs in a bimodal injection profile starting at (a) *t* = 0, showing (b) diffuse NIR emission at *t* = 26 s, (c) decreased intensity at *t* = 810 s, and (d–g) gradual endocytosis with SWCNT accumulation outlining cells and internal aggregates moving inward, with (h) the average NIR fluorescence intensity graph. Reproduced with permission from ref (82). Copyright 2008 American Chemical Society. (B) (a) Tracks of SWCNTs demonstrating corralled diffusion. (b) Representative 3D plots of SWCNT tracks demonstrating various types of diffusion and accompanying mean square displacement plots. (c) Three-dimensional tracking of SWCNTs showing intensity fluctuation during tracking. Reproduced with permission from ref (81). Copyright 2012 American Chemical Society.

The journey of real-time monitoring of cellular events commences with a pivotal understanding of the mechanisms governing the entry—endocytosis—and exit—exocytosis—of nanoparticles from cells. The dynamic tracking of these processes, particularly using NIR fluorescent SWCNTs, is considered a promising approach for advancing progress in this field. To this end, the Strano research group conducted two-dimensional single-particle tracking of DNA-dispersed SWCNTs *in vitro* for live monitoring of endocytosis, intracellular trafficking, and exocytosis (Figure 2A).^82^ As shown in Figure 2A(a–f), endocytosis progressed slowly during the first 100 min, with inward movement of aggregates observed around 125 min (Figure 2Ag). Figure 2Ah shows the total integrated intensity of NIR light collected by the detector throughout the entire duration of the *in vitro* tracking of SWCNTs, with arrows indicating the two peaks corresponding to the perfused pulses of SWCNTs across the stage. Importantly, this study marked the first-ever demonstration of SWCNT exocytosis enabled through detailed tracking. It was shown that the rates of endocytosis and exocytosis were nearly comparable with minor temporal offsets. Additionally, real-time tracking confirmed the intracellular accumulation and aggregation of the SWCNTs. This study not only opened new avenues for live monitoring of intracellular processes but also provided unprecedented concrete evidence for phenomena such as the exocytosis of SWCNTs.

Despite significant advancements in two-dimensional single-particle tracking of intracellular processes with SWCNTs, it is important to recognize that these methods face limitations in spatial and temporal resolution. These constraints arise from particle drift in the *z*-direction, moving them out of the focal volume, and in the *x*–*y* direction, taking them out of the field of view. To address these limitations, the Strano research group developed a strategy for three-dimensional tracking of SWCNTs in living cells using an orbital tracking microscope.^81^ Given that SWCNTs’ diffusion in water was too rapid for practical measurement using an orbital microscope with 32 ms/orbit, glycerol was chosen as the solvent to increase solution viscosity. At high viscosities, the slow rotation of SWCNTs allowed the use of rotational diffusion constants to calculate their lengths. Conversely, at low viscosities, translational diffusion constants were employed for this purpose. The three-dimensional motion of SWCNTs within HeLa cells was used to determine the active transport velocities and corral volumes. Importantly, the corral volume was estimated to range from 0.27 to 1.32 μm^3^, while the active transport velocity was calculated to be active transport velocity of 455 nm/s (ranging from 422 to 490 nm/s). The correlation between SWCNTs’ rotation and translation observed *in vitro* was utilized to estimate local apparent viscosities and SWCNT lengths (Figure 2 Ba–b). Additionally, endosomal aggregation of SWCNTs was observed 1.5 h after adding the SWCNT dispersion to the cell medium, as shown in the widefield channel (left panel, Figure 2Bc) and the integrated photoluminescence image from SWCNTs emission (right panel, Figure 2Bc). In the right panel, red arrows indicate SWCNT aggregation within the cells, while white arrows highlight aggregation surrounding the cells. Estimating parameters like corral volumes, generally not possible with two-dimensional tracking, clearly highlights the advantages of three-dimensional tracking of fluorescent nanoparticles in biology. This advanced method provides a comprehensive understanding of intracellular dynamics compared to the limited information on corral surfaces offered by two-dimensional tracking.

**Figure 3:**
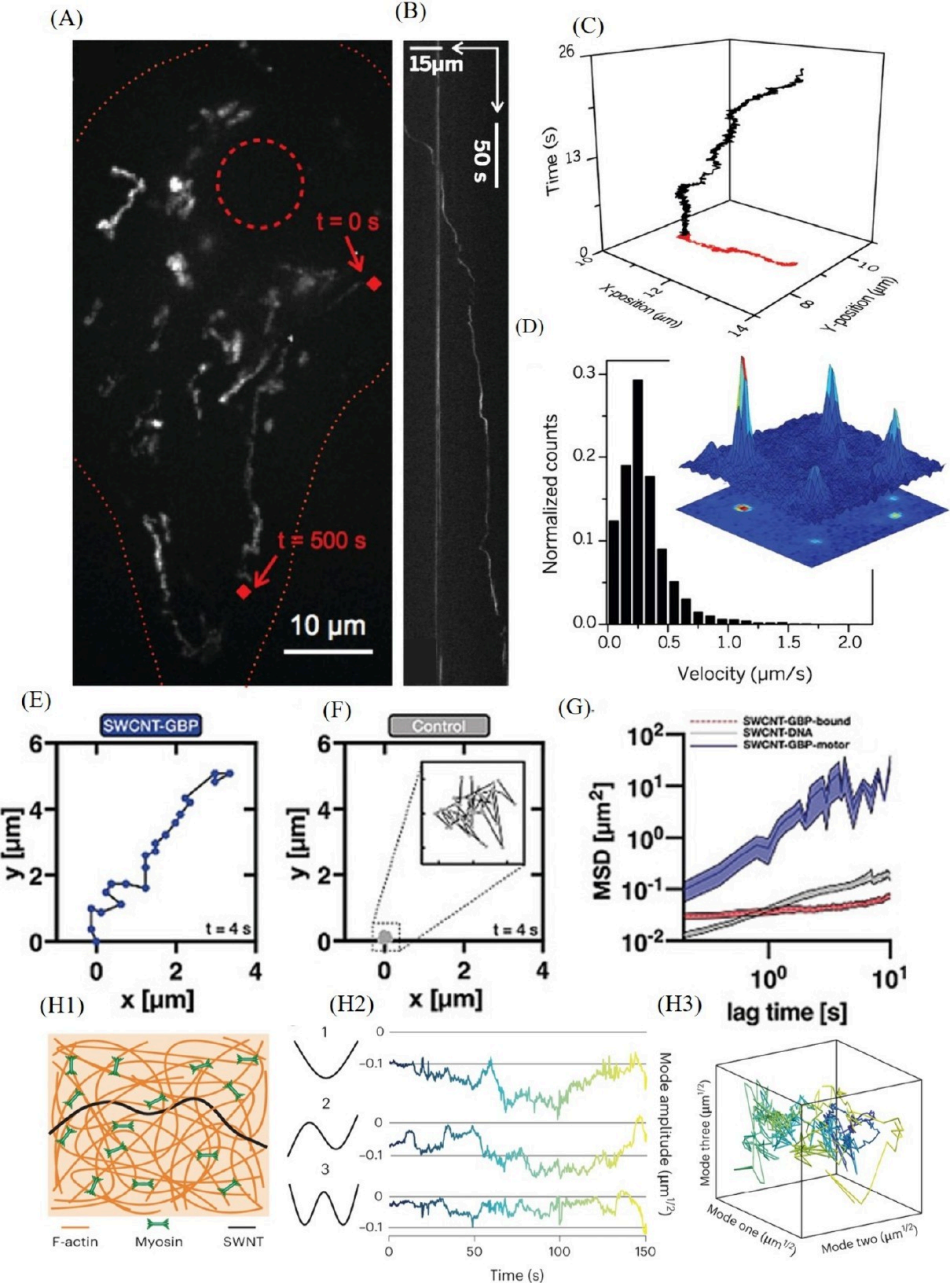
(A) Trajectories of SWCNT-labeled kinesin-1 motors (Kif5c) within a COS-7 cell. (B) Kymograph depicting the movement of a single SWCNT-labeled kinesin over a distance of approximately 40 μm. (C) Trajectory of a SWCNT-labeled kinesin observed within a cell with a frame interval of 5 ms. (D) Average velocity magnitude histogram of SWCNT-labeled kinesins computed over 2-s segments. Reproduced with permission from ref (83). Copyright 2014 American Association for the Advancement of Science (AAAS). (E) Real-time tracking of directional movement of a single Kin-5 motor in a *Drosophila* embryo, and (F) random movement of control DNA SWCNTs over a 4-s time span. (G) Mean square displacement plots comparing control DNA-SWCNTs with SWCNTs-GBP bound to static or active Kin-5-GFP. Reproduced with permission from ref (106). Copyright 2019 John Wiley & Sons. (H1) Schematic depicting the integration of SWCNTs into filamentous actin (F-actin) structures, subsequently interacting with myosin filaments. (H2) Analysis of the conformational dynamics of SWCNTs, illustrating their decomposition into the normal modes of a free filament alongside the time series of the corresponding normal mode amplitudes. (H3) Visualization of the multidimensional mode time series. Reproduced with permission from ref (108). Copyright 2023 Nature.

Furthermore, in a study by the Schmidt and MacKintosh research group, cytoskeletal dynamics were investigated using SWCNTs.^83^ Motor proteins, vital for cellular motion, serve as key indicators of the dynamics within cells. Thus, understanding the behavior of these motors within cells is crucial. In simple terms, motor proteins transport cargo along microtubules, akin to trucks carrying loads on roads.^105^ In this context, SWCNTs were bound to the endogenous kinesin-1 motor Kif5c, a known cargo transporter in cells, within cultured COS-7 cells. SWCNTs were suspended with DNA oligonucleotides and a Halo Tag to precisely bind them to full-length kinesins via covalent attachment. About 100 SWCNTs were introduced per cell, and individual SWCNTs were tracked for 90 min (Figure 3A–B). Furthermore, to capture shorter dynamics, imaging was conducted with a temporal resolution of 5 ms per frame (Figure 3C). The tracks of the moving SWCNTs displayed long, mostly straight runs, typical of kinesin-1 activity. The average velocity during these runs, calculated with a low-pass filter over 2-s intervals, was 300 ± 210 nm/s (Figure 3D). A single-molecule tracking algorithm facilitated the precise centroid position determination of SWCNTs, enabling quantitative assessment of intracellular motion ranging from milliseconds to hours. Live tracking of SWCNTs revealed an active random “stirring” regime as an intermediate mode of transport, distinct from thermal diffusion and directed motor activity. High-frequency motions were mainly thermally driven. At time scales above hundred milliseconds, nonequilibrium conditions prevailed, where in addition to directed transport along microtubules, another form of movement driven by a different motor protein, myosin, contributed to further randomization of transport. Live-tracking of cytoskeletal dynamics using SWCNTs opened avenues for monitoring active processes in cells, thereby enhancing our understanding of fundamental cellular processes.

The Kruss research group has pioneered the use of DNA-suspended SWCNTs conjugated with a green fluorescent protein (GFP) targeting nanobody (SWCNTs-GBP) to track the movement of Kinesin-5-GFP (Kin-5-GFP) motor proteins in *Drosophila melanogaster* embryos.^106^ These SWCNTs-GBP exhibited directional movement along microtubules with a mean velocity of approximately 1340 nm/s, thereby confirming their successful binding to Kin-5-GFP (Figure 3E). In contrast, a control set of DNA-suspended SWCNTs displayed random movement over the same time frame (Figure 3F). Furthermore, the analysis of the mean square displacements revealed distinct behaviors of SWCNTs-GBP depending on whether they were bound to static or active Kin-5-GFP motors (Figure 3G). The novelty of this study lies in the innovative fabrication of a nanomolecular hybrid capable of real-time monitoring of crucial, nonequilibrium biological processes.^107^ By providing new opportunities to study cellular dynamics, it enables the extraction of real-time information on active cellular processes, thus offering valuable insights into cellular behavior.

Furthermore, SWCNTs were employed as probes to track flow patterns in model actomyosin cortices with varying cross-link densities.^109^ The study revealed that these cortices autonomously adopt different nonequilibrium steady states. At lower cross-link densities, they exhibit symmetric, divergence-free, long-range flow patterns, whereas higher densities result in symmetry-breaking converging flow patterns. These findings provide valuable insights into how biological systems maintain stability and functionality through dynamic self-organization. Additionally, SWCNTs integrated into *Xenopus* egg extracts were used to explore nonequilibrium dynamics within complex cytoskeletal networks (Figure 3H1–H3).^108^ By monitoring of asymmetries in SWCNT motion, the study elucidated the dynamics of nonequilibrium activities across different scales. The method’s sensitivity to ATP/ADP ratios and network modifications offered insights into how microscopic interactions influence broader nonequilibrium behaviors in biological systems.^110^

### 3.3 At the Tissue Level

The hierarchical organization and specialization of cells contribute to the greater complexity of biological processes at higher levels of organization, including tissues and organs. Consequently, monitoring biological events in real-time within tissues offers invaluable insights into complex interactions and functional outcomes. This broader perspective facilitates the understanding of systemic reactions, intercellular signaling, and the convergence of numerous physiological mechanisms. Taking into account this panoramic viewpoint, diagnostics become more precise and treatments more effective, taking into account the organism’s complete health context.

In addition to photostability and biocompatibility, an essential criterion for a probe to effectively function within tissues and organs is an appropriate length-to-diameter aspect ratio.^111^ This ratio must facilitate penetration within multicellular ensembles. Furthermore, to provide real-time information about the local environment in tissues and organs, it is crucial for the probes to possess a suitable length and rigidity. These characteristics help moderate their diffusion rates to align with video-rate single-molecule imaging capabilities.^111^ Once stabilized with appropriately chosen corona phases, they should be rendered with suitable length and rigidity to feasibly allow their diffusion into a complex biological environment with varying viscosities.

**Figure 4:**
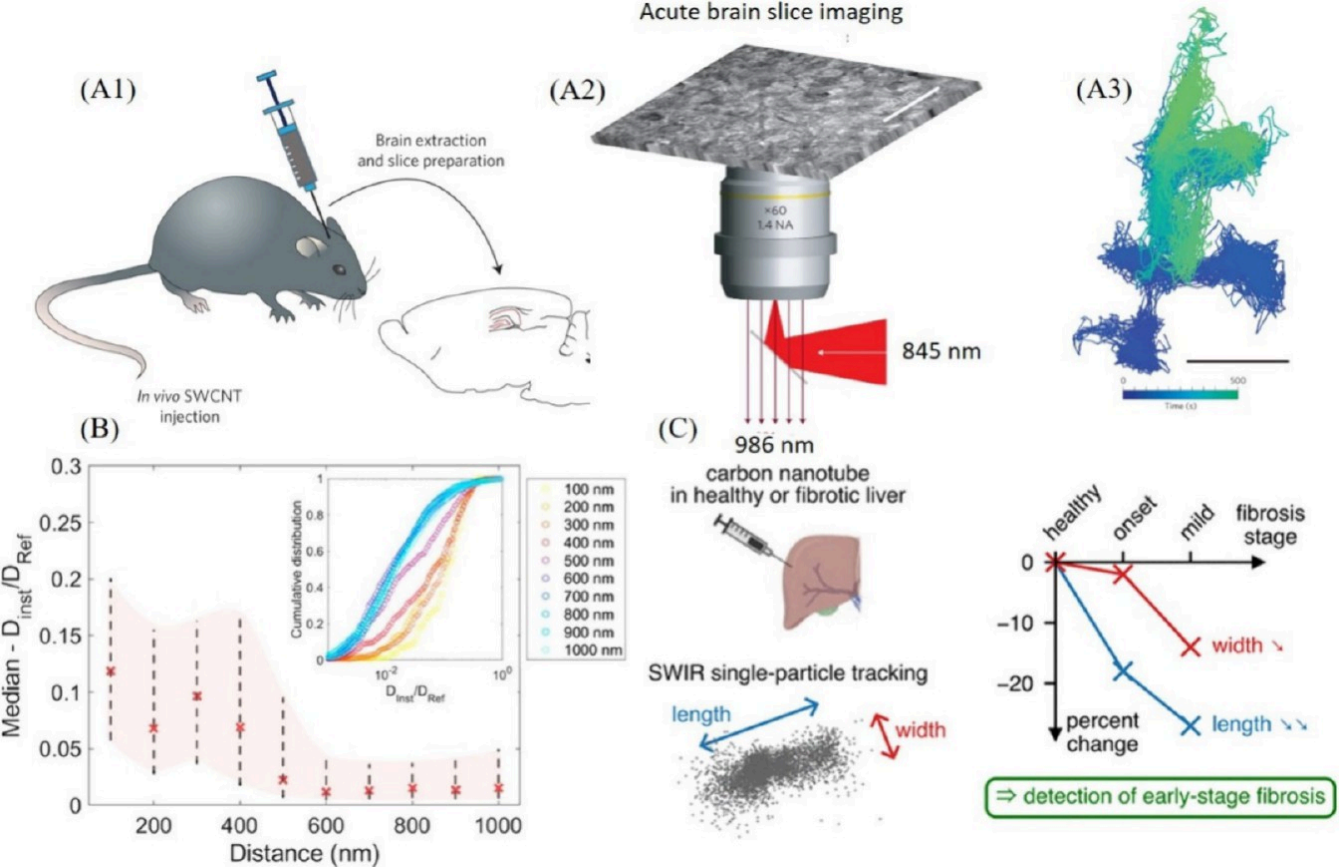
(A1) Schematic showing the introduction of SWCNTs into the lateral ventricles of young rats and their subsequent diffusion into the neocortex, (A2) imaging of SWCNTs in brain slices, and (A3) color-coded trajectory showing diffusion of single SWCNTs in ECS. Scale bar is 1 μM. Reproduced with permission from ref (111). Copyright 2017 Nature. (B) Median values and cumulative distributions (inset) of local diffusivities of SWCNTs measured in various coronal regions surrounding synapses. Reproduced with permission from ref (84). Copyright 2022 American Chemical Society. (C) Schematic illustrating the injection of SWCNTs into healthy and fibrotic liver environments. The tracking of SWCNTs in the short-wave infrared (SWIR) window provides real-time information about the progression and prognosis of liver fibrosis. Reproduced with permission from ref (85). Copyright 2024 American Chemical Society.

In this context, the Cognet research group reported the successful tracking of single SWCNTs within the extracellular space (ECS) in live brain tissue.^111^ Initially, (6,5) SWCNTs dispersed with phospholipid-poly(ethylene glycol) (PL–PEG) were injected into the lateral cerebroventricles of young rats. Subsequently, acute brain slices were prepared for inspection under a wide-field fluorescent near-infrared microscope. Following detailed investigations, particularly by following the trajectory of the SWCNTs, the dimensions and viscosity of the ECS could be estimated at various regions (Figure 4A1–A3). Intriguingly, a notable diversity of ECS dimensions reaching as low as 40 nm, alongside fluctuations in local viscosity values, could be observed. The viscosity of ECS was found to be 50 mPa s, which is 2 orders of magnitude greater than the viscosity of cerebrospinal fluid. This research is anticipated to enhance our understanding of the nanoscale ECS network. This is further expected to catalyze the development of innovative strategies for drug delivery.

In an allied vein, considering the crucial role of synapses in neuronal communication, the local environment of the ECS around synapses was also mapped using (6, 5) SWCNTs suspended with PL–PEG.^84^ Initially, synapses in brain slices were labeled using GFP-PSD95 lentivirus vectors. Subsequently, the local diffusivity of the SWCNTs was analyzed. Remarkably, the local diffusivity of the SWCNTs exhibited sharp variations at the submicron scale—specifically within 400 to 500 nm (Figure 4B, insert). It was observed that the SWCNTs experienced a 10-fold increase in diffusivity near synapses compared with regions farther away (Figure 4B). Detailed investigations revealed that the “juxta-synaptic” environment—the area adjacent to a synapse, where communication between neurons occurs—extends up to 500 nm from synaptic centroids. Importantly, the diffusivity of the SWCNTs was significantly higher in this region compared to the “non-juxta-synaptic” area. More intriguingly, the rates of synaptic transmissions were found to remain unaltered despite the presence of SWCNTs. Additionally, the dimensions of the ECS were estimated via dynamic tracking using SWCNTs, revealing that the local ECS dimensions were highly heterogeneous, with widths ranging from 50 nm to 1 μm and beyond. Finally, it was shown that the diffusion characteristics of the ECS juxta-synaptic region vary in response to neuronal activity, suggesting that this nanoenvironment could be involved in regulating brain function. The comprehensive study of the ECS surrounding synapses, conducted via real-time dynamic tracking of SWCNTs, is anticipated to significantly enhance our understanding of brain function and behavior.

Similarly, the alterations in the diffusivity of SWCNTs within the extracellular matrix were employed as tools to monitor the early stages of liver fibrosis.^85^ This progression is marked by modifications in the hepatic extracellular matrix. The Cognet research group aimed to determine if these changes could translate into differential diffusion of SWCNTs, potentially offering real-time prognostic information on liver fibrosis—especially at earlier stages compared to conventional methods such as Sirius red staining of collagen, a typical indicator of liver fibrosis. In line with previous studies, SWCNTs were suspended with PL–PEG. Fibrosis in mice was induced by standard protocols involving the injection of carbon tetrachloride (CCl_4_), and the successful induction of fibrosis was confirmed through a histopathological examination. Subsequently, SWCNTs were injected into the liver lobe, and imaging was possible even at distances of up to 1 cm from the injection sites. Notably, alterations in the interstitial matrix during the initial disease stages led to constraints in the exploration range of the nanotubes. During the initial stages of the disease, SWCNT mobility was remarkably restricted even in regions far from major vessels, giving real-time information about the early prognosis of fibrosis (Figure 4C). Interestingly, Sirius red, the current gold standard for diagnosis, did not exhibit collagen staining in these particular regions, thereby highlighting the high sensitivity of SWCNTs in diagnosing liver fibrosis. This unique study, facilitating early disease diagnosis through dynamic SWCNT tracking in complex biological environments, has the potential to revolutionize clinical research and significantly impact our understanding of health and disease.

### 3.4 At the Organism Level

The complexity in biological systems ultimately extends to an entire organism through hierarchical organization of structures and functions, beginning at the molecular level and culminating in a fully integrated, functioning organism. Therefore, real-time tracking of biological events at the organism level provides a comprehensive and physiologically relevant understanding of biological processes, which is essential for translating research findings into practical applications in medicine, pharmacology, and biology.

In this regard, SWCNTs have been extensively used to provide real-time information about biological processes occurring on the macroscopic scale. This approach was inspired by pioneering studies from the Weisman research group, which highlighted the use of SWCNTs for *in vivo* applications.^112,113^ However, for the successful utilization of SWCNTs, it is crucial to ensure that they do not undergo chemical degradation under extreme physiological conditions, such as variations in pH and ionic strength. This issue can be addressed by dispersing the SWCNTs with robust corona phases that are both biocompatible and do not compromise the photophysical properties of the SWCNTs.

**Figure 5:**
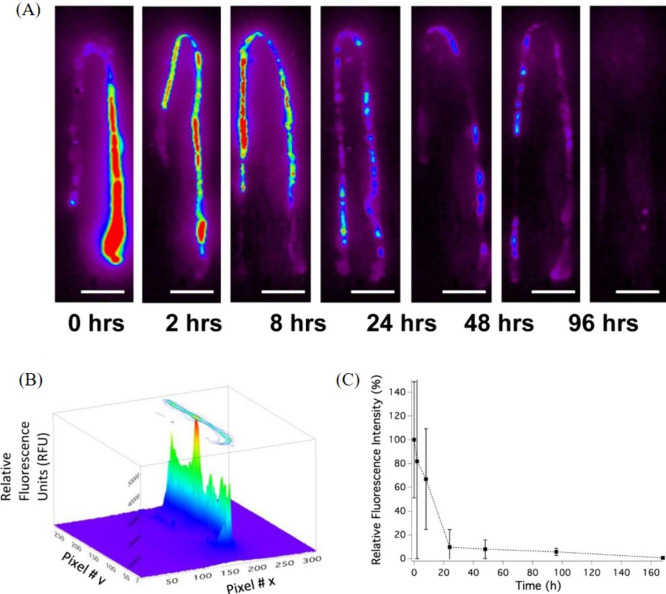
(A) NIR fluorescence images of fish intestines (administered with SWCNTs) over time show initially strong fluorescence postgavage, gradually decreasing with complete excretion taking 168 h. (B) Spatial fluorescence intensity mapping for semiquantification of image intensity. (C) Plot of intensity over time compared with quantified SWCNT burdens in fish intestines. Reproduced with permission from ref (88). Copyright 2014 American Chemical Society.

To this end, the Sabo-Attwood research group has tracked and quantified SWCNTs in an aquatic vertebrate model.^88^ Specifically, the distribution of SWCNTs in Fathead minnows (*Pimephales promelas*) was assessed using a NIR fluorescence imaging system over a period of 7 days. Initially, the toxicological effects of the SWCNTs were studied. Interestingly, although no apparent toxicity of the SWCNTs was observed, histopathological examinations revealed edema in the submucosa of the gastrointestinal tract (GI). The distribution analysis of SWCNTs indicated their predominant presence in the excised intestinal tissues, with no SWCNTs detected in other tissues examined, thereby eliminating the possibility of intestinal absorption of SWCNTs (Figure 5A–C). This study, which reports the *in vivo* tracking of SWCNTs to provide real-time information on the biodistribution of nanomaterials across tissues and organs, is crucial for developing safe and effective nanomedicines. Consequently, it significantly advances SWCNT-based therapeutics.

**Figure 6:**
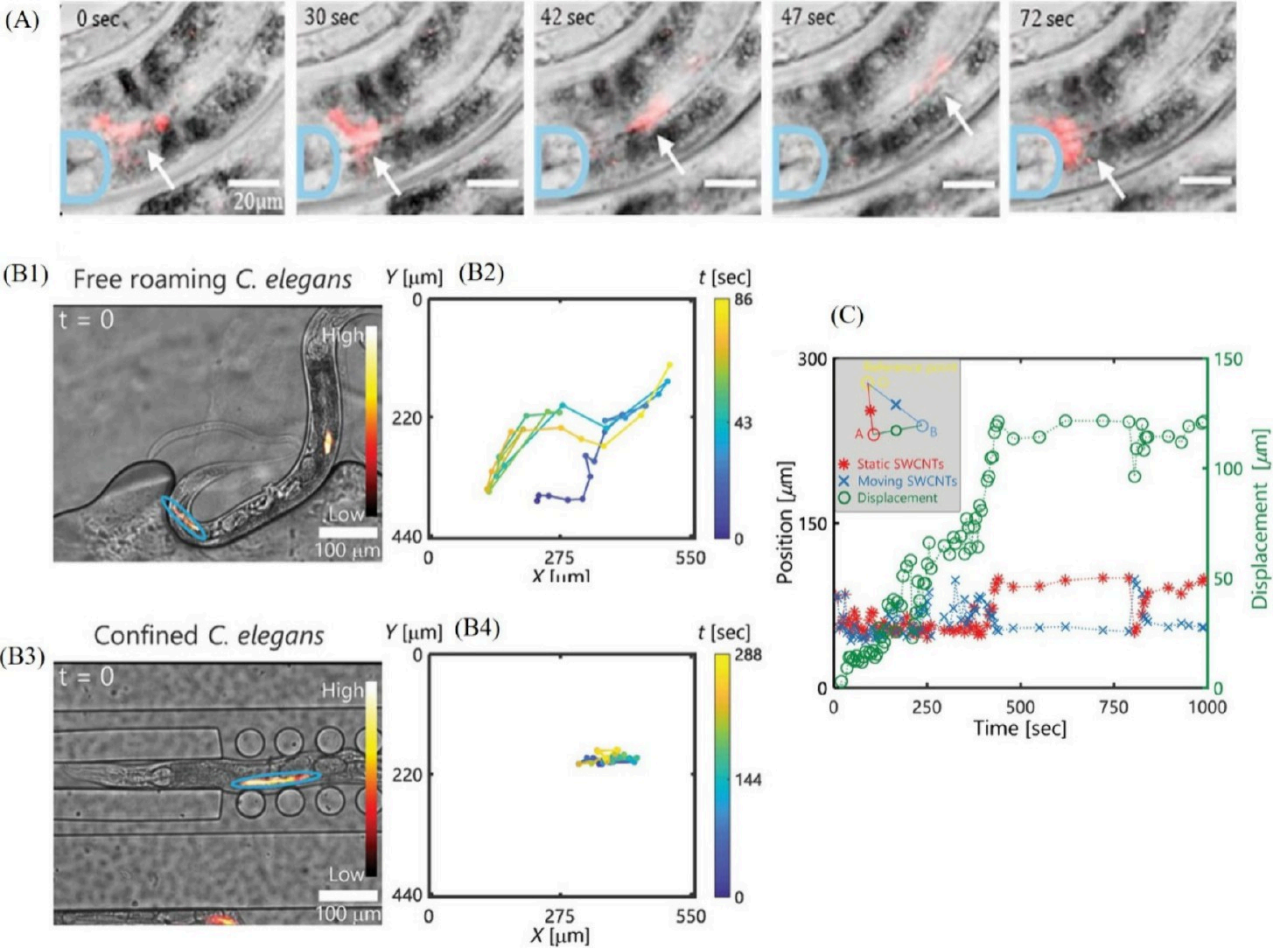
(A) Brightfield images overlaid with NIR fluorescence images showing the dynamics of SWCNTs in the worms. The white arrows are indicative of the time-dependent distribution and accumulation of the SWCNTs in the worms, and the area marked with blue highlights the terminal bulb. Reproduced with permission from ref (87). Copyright 2021 Elsevier. (B1) Bright-field images overlaid with NIR images, and (B2) tracking graphs of SWCNTs within a free *C. elegans*. (B3) Bright-field images overlaid with NIR images, and (B4) tracking graphs of SWCNTs within a confined *C. elegans*. (C) Quantitative analysis of the displacement of SWCNT clusters provides real-time insights into the worm’s digestion speed. Reproduced with permission from ref (86). Copyright 2024 John Wiley & Sons.

Moreover, real-time tracking of contraction and relaxation in the pharyngeal valve area at the anterior of the terminal bulb in *C. elegans* worms was demonstrated using SWCNTs as probes.^87^ Leveraging *C. elegans*, with a genome similar to humans, has proven invaluable for deciphering the prognosis of human diseases. Initially, the worms were incubated with DNA-SWCNTs, showing no apparent toxicity to the organisms. Subsequently, the real-time mobility and dynamics of SWCNTs within the worms were studied. This investigation was particularly feasible as the autofluorescence of the worms throughout the visible range did not interfere with the SWCNT fluorescence in the NIR spectral region where the primary acquisition was conducted. Notably, the SWCNTs were observed to traverse the pharynx and the pharyngeal valve into the lumen of the intestine, with clear localization after passing through the pharyngeal grinder at the anterior of the terminal bulb or within the intestine. Over time, the periodic distribution and accumulation of SWCNTs at the anterior of the terminal bulb was observed (Figure 6A). This phenomenon unveiled the pumping motion, i.e., muscle contraction of the pharynx, resulting in increased pressure in the terminal bulb and facilitating the passage of food through the pharyngeal-intestinal valve into the intestine. Additionally, the movement of SWCNTs within the intestine provided insights into the digestion process in the worms. Moreover, the time scale of GI contractions was quantitatively assessed by analyzing SWCNT fluorescence signal intensity, resulting in a contraction periodicity in the range of 10–20 s. This study, achieving real-time information about the contraction of the GI and feeding mechanisms in worms, opens new horizons in monitoring biological processes and lays the groundwork for advancements in clinical diagnostics.

An inherent challenge in real-time tracking of SWCNTs (and other probes) in live organisms is the ability to control their locomotion, often necessitating immobilization through anesthetics or the utilization of complex setups. Addressing this precise issue, the Bisker research group demonstrated spatiotemporal tracking of SWCNTs in confined *C. elegans* within a microfluidics setup to achieve real-time information on the feeding mechanism of the worms.^86^ The utilization of microfluidic settings for the real-time tracking of probes in *C. elegans* presents several advantages, including minimal animal stress, compatibility with imaging settings, high throughput readouts, and reusability. The authors have effectively demonstrated the benefits of live-tracking of confined *C. elegans* worms compared to free-moving worms, wherein free worms were observed to exit the field of view relatively quickly, thereby posing challenges for real-time tracking (Figure 6B1–B4). The study began with the internalization of SWCNTs into the worms through nutritional sources. The feeding mechanism in the worms was validated using SWCNTs suspended with two different types of corona phases—namely, DNA and PL–PEG. Interestingly, distinct nanotube varieties exhibited varied patterns of uptake, accumulation, detectability, and dynamics during the feeding process, attributable to their unique molecular compositions and optical properties. Interestingly, it was observed that a small cluster of SWCNTs detached from a larger cluster and moved through the worm’s digestive tract, while the larger cluster remained relatively stationary. The distance between the stationary source cluster and the moving cluster increased steadily up to 120 μm, which is approximately 13% of the average length of an adult hermaphrodite’s intestine, reflecting a digestion speed of about 8 μm per minute (Figure 6C). By introducing microfluidics, this study transcends the conventional limitations of live imaging of entire organisms while providing real-time insights into the feeding mechanisms of nematodes. Given their genetic similarities to humans, these findings hold promise for diagnostics and personalized medicine, offering real-time feedback on digestive mechanisms and paving the way for innovative therapeutic interventions.

## 4 Challenges and Future Outlook in Dynamic Tracking of Biological Processes Using SWCNTs

The studies discussed here emphasize the significant growth in the field of dynamic tracking of biological processes using NIR fluorescent SWCNTs, driven by active research in recent years. Nevertheless, considerable scope for further advancement in this domain. For example, exploring SWCNTs as probes for real-time biological monitoring with super-resolution microscopy represents an intriguing avenue for future research. However, using SWCNTs as probes in super-resolution microscopy also presents significant challenges, stemming from two main factors. First, SWCNTs emit light at longer wavelengths compared to visible spectrum probes, thereby potentially lowering the resolution due to the diffraction limit. Second, the high aspect ratio of SWCNTs contradicts the typical assumption in super-resolution microscopy that probes act as point-like emitters. Nonetheless, recent studies indicate promising approaches such as super-resolution radial fluctuation (SRRF), mean-shift super resolution (MSSR), and deblurring by pixel reassignment (DPR) algorithms, as well as deep learning with convolutional neural networks, to enhance the spatial resolution of SWCNT images.^114−116^ With these advancements, real-time tracking of biological processes using SWCNTs can achieve improved spatiotemporal resolution and enable quantitative analysis, marking a significant step forward in the field of super-resolution microscopy.^117,118^

In another context, the NIR emission of SWCNTs is crucial to their effectiveness as probes for tracking biological processes due to its alignment with the biological transparency window. However, this same characteristic—the emission in the NIR range—can also restrict their broader use in real-time biological tracking, as it requires specialized imaging technologies. To address this challenge, employing chirality-separated SWCNTs that emit NIR light within a range detectable by widely available silicon-based detectors could significantly enhance their accessibility and practical utility in various biomedical applications.^119,120^ Notably, the separation of chirality-pure samples has become increasingly achievable through advanced techniques such as aqueous two-phase extraction,^121−124^ DNA-based separation techniques,^125^ and polymer-based single-chirality selective suspension of SWCNTs.^56,77,126^ By harnessing the availability of these chirality-pure SWCNTs and using them to track biological events in a spectral region where silicon-based detectors offer high sensitivity, we could greatly expand the potential of SWCNTs for real-time monitoring of biological processes could be greatly expanded.

Furthermore, real-time tracking of SWCNTs within *in vivo* models could provide valuable insights into the progression of degenerative diseases such as Alzheimer’s. Similarly, observing cell division in real-time using SWCNTs under the influence of potent carcinogens could illuminate the timing and mechanisms of cancer progression. Additionally, SWCNT-assisted real-time monitoring of cholesterol deposition in blood vessels and arteries could offer insights into the progression of atherosclerosis. Therefore, real-time tracking of SWCNTs within *in vivo* models to understand disease progression has the potential to significantly impact human health and the control of diseases.

Another promising avenue to fully leverage the potential of SWCNTs in the dynamic tracking of biological processes is through multiplexed tracking. This approach could involve tracking multiple organelles within cellular environments or monitoring various biological processes across different organs in an organism. Such multiplexed tracking could be achieved by utilizing SWCNTs of different chiralities, with each chirality possessing distinct excitation-dependent emission properties. By employing appropriate excitation and emission filters, this strategy may enable the simultaneous tracking of different processes in distinct compartments of the biological species.

## 5 Conclusion

In this review, we explored in detail the studies that utilized SWCNTs as probes to monitor active biological processes originating at the molecular level up to the whole-organism level. We have highlighted the essential properties of SWCNTs that make them suitable for such studies. Further, we have organized the studies into four hierarchical levels—(i) molecular, (ii) cellular, (iii) tissue, and (iv) organism—to illustrate the progressive application of SWCNTs for monitoring biological processes across different biological scales. For each scale, we discuss the importance of tracking biological processes at each level, outline the general principles of using SWCNTs for tracking, offer a detailed yet concise discussion of the studies, including quantitative data, and highlight the unique advantages of each study. Additionally, we identified the critical features of these studies that have driven advancements in this field, showcasing how SWCNTs have enabled real-time tracking and provided valuable insights into complex biological systems. Furthermore, we have proposed various new directions for exploration and suggested potential ways to achieve these advancements. The possibilities for using SWCNTs to track biological events in real-time within intricate environments are immense. Ongoing research in this area is poised to lead to significant breakthroughs and innovative applications.
